# Early career innovators shaping the future of bioengineering and translational medicine

**DOI:** 10.1002/btm2.70153

**Published:** 2026-06-09

**Authors:** Elizabeth Nance

**Affiliations:** ^1^ Department of Chemical Engineering University of Washington Seattle Washington USA

Advances in bio and chemical engineered technologies that are translationally relevant are increasingly driven by investigators who, early in their independent careers, cross disciplinary boundaries, embrace risk, and pursue clinically meaningful impact alongside fundamental discovery. This Early Career Innovator (ECI) Special Issue of Bioengineering & Translational Medicine highlights a cohort of emerging leaders developing innovative technologies to address pressing challenges in human health. Collectively, these contributions exemplify the mission of BioTM and align with broader efforts by AIChE and the Society for Biological Engineering (SBE) to elevate the voices and work of the next generation of innovators in the field.

The authors featured in this issue showcase a shared commitment to translation: transforming advances in materials science, biofabrication, synthetic biology, and mechanobiology into technologies that can ultimately benefit patients. At the same time, they represent a remarkable diversity of approaches, disease targets, and institutions. Together, their work offers a snapshot of where the field is headed and how early career investigators are helping to define its trajectory.

Several contributions in this issue focus on the design and engineering of biomaterials and delivery systems for improved therapeutic performance. Mike Mitchell, Associate Professor of Bioengineering at the University of Pennsylvania, brings forward work at the intersection of nanomedicine and immunoengineering, where rationally designed carriers are being developed to overcome long‐standing barriers in drug and vaccine delivery. Mukalel et al. offer a potential translational path toward solid tumor immunotherapy via a bioinspired mRNA lipid nanoparticle delivery platform that enables functional CAR macrophage engineering with minimal immunogenicity.^1^ Amir Sheikhi, Associate Professor and Dorothy Foehr Huck and J. Lloyd Huck Early Career Chair in Biomaterials and Regenerative Engineering at Pennsylvania State University, explores how molecular‐ and nano‐scale control over material properties can be leveraged to dynamically interact with biological systems. Specifically, the Sheikhi lab developed porous stable gelatin methacryloyl microgels that form robust granular hydrogel scaffolds at physiological temperatures. These scaffolds comprise interlinked microgels that promote cell infiltration, vascularization, and metabolite transport through their interconnected macropores, which could provide in situ regeneration of irregular soft tissue defects.^2^ Jessica Larsen, Carol and John Cromer '63 Family Endowed Associate Professor of Chemical and Biomolecuar Engineering at Clemson University, demonstrates an approach that engineers a novel computed tomography (CT) contrast agent to enhance the usability of CT for tumor imaging. The Larsen lab created gold‐loaded polymersomes with high CT contrast in vitro and in vivo, providing an alternative to standard contrast agents that can also increase visualization of tumor sites, allow for timely detection, and enable identification of tumor margins via CT.^3^

Understanding and exploiting cell–material and cell–environment interactions is another unifying theme across the issue. Katharina Maisel, Associate Professor of Bioengineering at the University of Maryland, examines how transport barriers in tissues influence therapeutic delivery and response, offering insights that inform the design of smarter, more effective interventions. Jankowski et al. developed a low‐cost, efficacious, methyl cellulose‐based ethanol gel to topically treat low‐grade cervical dysplasia, which addressed the critical need for an accessible treatment option for women in low‐ and middle‐income countries.^4^ Brittany Taylor, Assistant Professor of Biomedical Engineering at the University of Florida, focuses on how engineered systems can be used to interrogate cellular behavior relevant to disease progression and treatment. Her work generates a representative 3D tendon model to study tendinopathy progression that reveals dynamic changes in tendon cell behavior and extracellular vesicle (EV) cargo linked to fibrosis and inflammation.^5^

Other contributors showcase the growing impact of bioinspired design and mechanobiology in translational research. Ritu Raman, Eugene Bell Career Development Assistant Professor of Mechanical Engineering at Massachusetts Institute of Technology, integrates principles from biology, materials science, and robotics to create living and hybrid systems that respond to their environment, opening new possibilities for adaptive therapeutics and soft bioactuators. The Raman lab leverages optogenetic techniques and open‐source hardware to develop a minimally invasive high‐throughput muscle exercise assay and generates RNA sequencing datasets in response to several muscle training regimens, which can inform practical next steps for cultivating mature skeletal muscle models.^6^ Stefano Menegatti, Professor & University Faculty Scholar in Chemical and Biomolecular Engineering at North Carolina State University applies engineering principles to the design of functional biomolecules and interfaces, bridging fundamental structure–function relationships with translational applications in diagnostics and therapy. Menegatti's team addresses a critical biomanufacturing bottleneck by introducing peptide‐based affinity resins and affinity membranes specifically designed for the capture of Lentiviral vectors.^7^ Their platforms demonstrate high binding capacity, productivity, and robust removal of host cell contaminants, enabling a streamlined, four‐step purification process with clinically relevant yields and purity.

The issue also highlights innovation in micro‐ and mesoscale systems, where creative engineering enables new modes of biological interrogation and intervention. Saad Bhamla, Associate Professor of Chemical and Biomolecular Engineering at Georgia Institute of Technology, exemplifies this approach by developing low‐cost, accessible technologies with applications ranging from global health diagnostics to mechanistic studies of living systems. Bhamla's team engineered portable, multi‐pulse piezoelectric electroporators for carrier‐free intradermal delivery of naked mRNA and plasmid DNA to enhance in vivo expression of reporter proteins.^8^ Gregg Duncan, Associate Professor of Bioengineering at University of Maryland, shows the integration of advanced fabrication and analytical techniques to better model physiological environments, enabling more predictive platforms for translational research. Duncan et al. uncover immune‐mediated mechanisms and design strategies to enhance nanoparticle delivery to tumors via PEGylation, which could be readily integrated into clinically relevant nanoparticle delivery systems.^9^

Finally, the importance of stem cell engineering, developmental biology, and regenerative medicine is reflected in contributions from Marissa Wechsler, Assistant Professor of Biomedical Engineering at the University of Texas at San Antonio, and Samira Azarin, Associate Professor of Chemical Engineering at the University of Minnesota. Their work leverages biomaterials, biophysical cues, and systems‐level design to control cell fate and function, advancing strategies for tissue regeneration and disease modeling with clear paths toward clinical translation. Wechsler et al. engineer a reactive oxygen species scavenging‐poly(propylene sulfide) nanoparticle system that incorporates Pluronic F‐127 and sucrose monolaurate to reduce inflammation and avoid an additional immune response. This work shows the promise of a treatment for chronic inflammatory diseases.^10^ The Azarin lab found that biophysical fragmentation of tumor cells induced by irreversible electroporation (IRE) plays a central role in shaping downstream immune responses by enhancing antigen presentation and promotes robust T‐cell activation compared to thermal and cryoablation approaches.^11^ This work provides actionable guidance for clinicians and device developers that tuning IRE treatment parameters to maximize cellular fragmentation may improve systemic anti‐tumor immunity and synergize with immunotherapies.

The articles in this ECI Special Issue demonstrate that early career investigators are not only advancing technical innovation but also shaping the future of translational bioengineering. Many of these researchers emphasize scalability, manufacturability, accessibility, and patient‐centered design; these are essential considerations for moving technologies from the laboratory to the clinic. Just as importantly, they reflect a generation of scientists trained to work fluently across disciplines, collaborating with clinicians, engineers, scientists, and stakeholders to accelerate impact.

This issue would not be possible without the dedication of the contributors, reviewers, and editorial team. We hope readers find this collection both informative and motivating, and that it provides a window into the future of translational bioengineering that is being actively built today by the early career scientists featured here.

## AUTHOR CONTRIBUTIONS

**Elizabeth Nance:** Conceptualization; writing – original draft; writing – review and editing.

## CONFLICT OF INTEREST STATEMENT

The author declares no conflicts of interest.

## Data Availability

Data sharing not applicable to this article as no datasets were generated or analyzed during the current study.
